# Arrhythmias in Patients with Cardiac Amyloidosis: A Comprehensive Review on Clinical Management and Devices

**DOI:** 10.3390/jcdd10080337

**Published:** 2023-08-05

**Authors:** Alexandros Briasoulis, Christos Kourek, Adamantia Papamichail, Konstantinos Loritis, Dimitrios Bampatsias, Evangelos Repasos, Andrew Xanthopoulos, Elias Tsougos, Ioannis Paraskevaidis

**Affiliations:** 1 Medical School of Athens, National and Kapodistrian University of Athens, 15772 Athens, Greece; chris.kourek.92@gmail.com (C.K.); adamantiapm@gmail.com (A.P.); lork21994@gmail.com (K.L.); bampatsiasdimitris@gmail.com (D.B.); v_repasos@hotmail.com (E.R.); iparask1@yahoo.gr (I.P.); 2 Department of Cardiology, University Hospital of Larissa, 41110 Larissa, Greece; andrewvxanth@gmail.com; 3 Department of Cardiology, Hygeia Hospital, 15123 Athens, Greece; cardio@tsougos.gr

**Keywords:** cardiac amyloidosis, arrhythmias, atrial fibrillation, ventricular arrhythmias, devices, clinical management

## Abstract

Cardiac amyloidosis (CA) is a rare but potentially life-threatening disease in which misfolded proteins accumulate in the cardiac wall tissue. Heart rhythm disorders in CA, including supraventricular arrhythmias, conduction system disturbances, or ventricular arrhythmias, play a major role in CA morbidity and mortality, and thus require supplementary management. Among them, AF is the most frequent arrhythmia during CA hospitalizations and is associated with significantly higher mortality, while ventricular arrhythmias are also common and are usually associated with poor prognosis. Early diagnosis of potential arrythmias could be performed through ECG, Holter monitoring, and/or electrophysiology study. Clinical management of these patients is quite significant, and it usually includes initiation of amiodarone and/or digoxin in patients with AF, potential electrical cardioversion, or ablation in specific patients with indication, as well as initiation of anticoagulants in all patients, independent of AF and CHADS-VASc score, for potential intracardiac thrombus. Moreover, identification of patients with conduction disorders that could benefit from prophylactic pacemaker implantation and/or CRT as well as identification of patients with life-threatening ventricular arrythmias that could benefit from ICD could both increase the survival rates of these patients and improve their quality of life.

## 1. Introduction

Cardiac Amyloidosis (CA) occurs when amyloid fibrils accumulate within the cardiac tissue, which is a condition that leads to progressive heart failure (HF), depending on the stage and the form of CA. Arrhythmias may be common in CA, and it is critical to identify them promptly because their effect is detrimental if left untreated. Heart rhythm disturbances in CA appear in all possible forms, such as supraventricular arrhythmias, conduction system disease, or ventricular arrhythmias, and they play a major role in CA morbidity and mortality [1].

Heart rhythm disorders in CA are consistent with disease progression both in terms of severity and clinical impact; hence, identifying them amidst early-stage disease management is vital for developing an appropriate treatment plan concerning pharmacological or invasive options [1,2]. Despite the acknowledged severity and clinical importance of arrhythmias in CA patients, more intensive research is necessary to fully understand their underlying causes. The aim of this review is to describe the most frequent arrhythmias in patients with cardiac amyloidosis, and to present diagnostic methods and clinical challenges in their treatment.

## 2. Cardiac Amyloidosis and Arrhythmias

Structural heart disease, such as CA, is related to a lower threshold for developing arrhythmias [3]. Their prevalence in this population varies depending on the CA subtype. According to the existing literature, patients with the amyloid light chain (AL) subtype of CA have a higher propensity to develop most of these arrhythmias than those with transthyretin amyloidosis (ATTR-CA) [4]. In AL CA, arrhythmias are present in up to 60% of patients, while in ATTR CA, the prevalence of arrhythmias ranges from 20% to 40% [4].

Among arrhythmias in patients with amyloidosis, AF and ventricular arrhythmias are the most frequent, and they may lead to an increase in both hospital mortality rates and length of stay [5,6]. In fact, ventricular arrhythmias are associated with poor prognosis, especially in AL cardiac amyloidosis [5,6]. Sudden cardiac death (SCD) is another significant cause of increased mortality with poor prognosis and a low 5-year survival in these patients [5,6].

Due to the low end-diastolic volume and the reduced stroke volume, patients with amyloidosis present a higher heart rate in order to compensate for their low cardiac output, and, as a result, they cannot usually tolerate beta-blockers [7].

Primary systemic AL amyloidosis patients are prone to atrioventricular and intraventricular conduction delays. These disorders are observed more commonly where there is cardiac involvement (27.5%) compared to its absence (16.5%) [8]. Abnormalities such as Left Bundle Branch Block (LBBB) (18.0% vs. 5.2%) and Incomplete Right Bundle Branch Block (iRBBB) (5.8% vs. 1.7%) are more prevalent in individuals with cardiac involvement [8]. Both Right Bundle Branch Block (RBBB) and Left Axis Deviation (LAH), however, exhibited no significant difference between the two groups. Results from 12-lead ECG recordings in patients showing conduction arrhythmias demonstrated widened PQ, QRS, QT, and QTc intervals. Additionally, cardiac biomarkers, such as B-type natriuretic peptide (BNP) and N-terminal pro-BNP (NT-proBNP), are substantially higher in those with intraventricular conduction delays [8]. These findings allude to severe cardiac involvement, the thickening of ventricular walls, impaired systolic and diastolic functions, and increased mortality, and they are associated with higher levels of cardiac biomarkers. It can therefore be discerned that for the efficient diagnosis and treatment of cardiac AL amyloidosis, BNP and NT-proBNP, along with being able to predict the degree of cardiac involvement and associated mortality, play an important role [8].

Heart rhythm frequently depends on the subtype of amyloidosis. For instance, AF is more common in ATTR compared to AL amyloidosis [9]. Other common features of ATTR patients are slow ventricular response, AV block, or intraventricular delays [9]. On the contrary, patients with AL amyloidosis present sinus rhythm and display a low voltage pattern at diagnosis [9]. These facts could lead us to the conclusion that the ATTR subtype behaves as progressive cardiomyopathy characterized by slow amyloid deposition within the atria, the ventricles, and the conduction system, while AL imitates acute myocarditis with early symptom onset and rapid disease progression to end-stage heart failure due to the toxic effects of AL chains [9].

## 3. Types of Arrhythmias

### 3.1. Atrial Fibrillation and Atrial Flutter

Atrial fibrillation (AF) is the most common arrhythmia in CA patients, with a prevalence ranging from 16 to 80% depending on the CA subtype and stage [10]. AF is more common among ATTR patients, while AL patients more often present with sinus rhythm with low voltages [9]. AF or AT is common in CA, especially as the disease progresses, with up to 70% of patients with ATTRwt CA having AF [11]. Left atrial voltage mapping shows significantly lower voltages in amyloid patients compared with an age-matched patient population with persistent AF [12].

Longhi et al. concluded that AF was present in 38% of their study patients at the time of diagnosis and that the incidence of AF increased with disease duration [10]. Sachis et al. found that the incidence of AF was 15% person/years, and that 44% of patients had a previous history of AF at the time of their diagnosis [13]. During follow-up, 36% of patients without a history of AF at diagnosis developed AF. However, AF did not impact all-cause mortality or cardiovascular mortality in CA patients [13].

Due to the high amyloid protein deposition, the left atrium (LA) presents significant structural and electrical remodeling, leading to chronic elevation of LA pressures, dilation, and dysfunction [7,14]. As a result, the prevalence of AF is higher in amyloidosis with increased mortality rates and length of stay [7,14]. Moreover, due to AF, thromboembolic events are also frequent in these patients [7,14]. AL amyloidosis as a single unity may increase thrombogenicity and thromboembolism without the presence of AF [7,14].

All AF subtypes may be present in CA. Donnellan et al., in a study of ATTR-CA, found that 45% of the included patients had paroxysmal AF, 27% had persistent AF, 15% had longstanding persistent AF, and 13% had permanent AF [15]. Patients with AF were older (78 ± 9 years vs. 73 ± 11 years), and they had larger left atrial diameters (4.7 ± 0.7 cm vs. 4.4 ± 0.8 cm) [15]. AF occurred more frequently in patients with ATTRwt (76%) compared to those with ATTRmut (54%) [15]. In a recent cohort of 18 CA patients, all patients presented with SVTs, with 17 having persistent AF or AT [16]. Sachis et al. showed that the AF subtype was not associated with all-cause mortality in CA patients, but that those with permanent AF had a trend towards a higher CHA2DS2-VASc score compared to those with non-permanent AF [13].

There are several risk factors for AF, including male gender, NYHA class III-IV, and renal insufficiency [10]. Patients with CA and arrhythmia were more likely to be older and male, and they had a higher frequency of co-morbidities, including HF, valvular heart disease, previous MI, peripheral vascular disease, pulmonary hypertension, and CKD [5].

Rocken et al. investigated the relationship between isolated atrial amyloidosis (IAA) and persistent AF in heart surgery patients [17]. The results showed that IAA is more common in older patients, women, and those undergoing mitral valve replacement. The presence of IAA was correlated with patient age, P-wave duration, and type of heart surgery, but not with NYHA functional class or ejection fraction (EF). Thus, IAA affects atrial conduction and increases the risk of AF, and the occurrence of IAA depends on age and is related to pathological conditions, such as valve diseases. The amount of amyloid correlated inversely with the degree of interstitial fibrosis, which suggests that patients with IAA may not benefit from treatment with ACE inhibitors to reduce the amount of atrial fibrosis. The study provides evidence that IAA is associated with AF, highlighting the importance of early detection and management of IAA in patients with cardiac conditions [17].

The clinical impact of AF in CA patients is significant. It is associated with a high thromboembolic risk, since 33% of autopsy cases of CA had intracardiac thrombi, which may be due to the combination of hypercoagulability, endomyocardial disruption, and endothelial dysfunction caused by amyloid deposition [18]. A recent study, which analyzed a population-based sample of 5585 CA-related hospitalizations in patients aged over 18 with or without arrhythmias, found that 36.1% of CA hospitalizations had concurrent arrhythmias, with AF being the most frequent arrhythmia observed (72.2%) [5]. Transthyretin cardiac amyloidosis is associated with increased risk of intracardiac thrombus. In general, current recommendations for anticoagulation in nonvalvular AF are based on a risk−benefit ratio that relies on the CHADS-VASc score [19]. However, Donellan et al. found no association between the CHADS-VASc score and LAA thrombus in subjects with ATTR-CA [20]. Therefore, systemic anticoagulation therapy should be considered in ATTR-cardiac amyloidosis patients with AF regardless of their CHADS-VASc score [20].

Atrial flutter (AFL) is another type of supraventricular arrhythmia that can occur in cardiac amyloidosis and that may increase the risk of stroke and HF [5,11,19].

### 3.2. Ventricular Arrhythmias

Ventricular arrhythmias (VA), such as ventricular tachycardia (VT) and ventricular fibrillation (VF), are less common in CA patients, but they are associated with a poor prognosis. They are more frequently observed in AL-CA, mainly due to its steep downward path following the onset of HF [18]. Nonetheless, recent evidence suggests that VAs may represent a frequent competing cause of death in both AL and ATTR. In a cohort of 5585 patients hospitalized for CA, 36% had concurrent arrhythmias, and VT was the second most common arrhythmia identified (14.9%), after AF (72.2%) [6]. The most frequent VA identified is VT, with a prevalence ranging from 3.5% to 28% [21]. Its presence can be life-threatening and requires immediate management. According to Palladini et al., patients with AL-CA can develop complex VA, couplets, and NSVT [22]. The prevalence of these arrhythmias was found to be 57%, 29%, and 18%, respectively. It is important to note that the significance of abnormal rhythms documented during monitoring but unaccompanied by symptoms is uncertain in virtually all heart diseases, including AL amyloidosis. Even VT can be seen occasionally in patients who subsequently remain asymptomatic during many years of follow-up [22].

According to Goldsmith et. al., VAs and AAs are common in CA patients following stem cell transplantation (SCT), and poor cardiac output is closely linked to severe VA [23]. The most frequent arrhythmia observed during peri-transplantation was NSVT. The diagnosis and treatment of life-threatening arrhythmia is possible by ongoing telemetry monitoring, which helps to ensure patient safety [23].

Multiple potential mechanisms can account for the occurrence of VAs in the CA population, including abnormal autonomic balance, reentry circuits, and myocardial ischemia due to amyloid infiltration of capillaries [24]. VF may be caused by PVC originating from the Purkinje system. However, the precise cause of VF arising in CA is not well understood [18].

The prognostic role of NSVT in CA is controversial, with some studies suggesting their association with SCD. NSVT was found in 27% of patients with AL-CA in a study by Dubrey et al. [25]. Nonetheless, recent evidence suggests that the impact of VAs on CA patients may have been undervalued, and they may represent a relevant competing cause of death in both AL and ATTR [6]. In CA patients undergoing stem cell transplantation (SCT), the prevalence of NSVT ranges from 5 to 27% with routine monitoring and 100% during SCT. In a study of patients with CA and ICDs in situ, VT/VF occurred in 27% of patients, with inappropriate shocks being uncommon and occurring in only 4.4% of patients [3].

The presence of VAs is associated with a higher risk of SCD and overall mortality. In a study of patients with CA and VT/VF, the 1-year mortality rate was 50%, with SCD accounting for 60% of deaths [26]. The presence of NSVT has also been associated with an increased risk of SCD [24].

### 3.3. Conduction System Disorders

Heart block is another type of arrhythmia that could occur in cardiac amyloidosis [11,19]. It can cause dizziness, fatigue, and fainting. Complete heart block can lead to agonal bradycardia, syncope, or even SCD. The prevalence of the need for pacemaker implantation at diagnosis ranges between 8.9% and 10% [6]. From an electrophysiological standpoint, patients with CA present a prolonged His-ventricle (HV) conduction interval compared to patients without CA. The HV conduction delay is more profound in ATTR than in AL [6]. The presence of these conduction abnormalities is associated with a higher risk of adverse outcomes, including SCD [27,28]. In particular, advanced AV block has been demonstrated to be associated with reduced left atrial contractile function in patients with ATTR CA [29].

Another particular electrophysiological characteristic in CA patients is the possible evidence of narrow ECG QRS complexes despite demonstrated prolonged H-V intervals, likely due to equal and homogeneous infiltration of both bundle branches and the distal conduction system. Prolongation of the H-V interval is more common in ATTR- than AL-CA, and it is associated with a higher risk of complete AV blocks and SCD due to electromechanical dissociation [24]. This may represent diffuse amyloid infiltration of the bundles, creating equal delays in both the right and left branches, yielding a disproportionately narrow QRS. According to a study by Reisinger et al. [30], all 25 patients with CA had abnormal 12-lead ECGs, and 23 patients had abnormally prolonged HV intervals, with a mean HV interval of 79 ± 18 ms (range 50 to 110). Marked HV prolongation (≥80 ms) occurred in 12 patients, 6 of whom had an interval ≥100 ms. Rapid incremental atrial pacing resulted in a block below the His bundle in only one patient. This study suggests that disturbances in the His-Purkinje system are common in patients with cardiac amyloidosis, and marked prolongation of the HV interval is often seen, even in the presence of a narrow QRS complex [16,30].

Conduction abnormalities, such as AV blocks, are associated with increased disease severity and lower survival rates in AL amyloidosis [1,8]. In addition, conduction delays are associated with higher LV wall thickness, indicating a potential relation between the amount of cardiac amyloid deposition and conduction abnormalities in AL amyloidosis [8].

### 3.4. Sudden Cardiac Death

Sudden cardiac death (SCD) is a devastating consequence of CA, and it can result from a variety of arrhythmias, including VAs, advanced AV block, and pulseless electrical activity. The incidence of SCD in AL CA patients without evidence of HF is estimated to be between 15% and 23%, making it a significant contributor to mortality in this population [31,32]. Arrhythmic SCD is far more common in AL cardiomyopathy, with elevated NT-pro-BNP and troponin levels, diastolic dysfunction on echocardiography, and the extent of extracellular volume and late gadolinium enhancement on MRI serving as adverse prognostic indicators [33]. Electromechanical dissociation is thought to be the most common cause of SCD in CA patients.

Syncope and SCD are common in CA patients, and a substantial proportion of CA patients die due to severe bradycardia with slow, wide-QRS escape rhythms or electromechanical dissociation rather than VAs. In a retrospective cohort of 280 patients with CA, patients with AF had a higher risk of death compared to those without AF (HR 1.8) [34]. In terms of VAs, the authors report that NSVT and sustained VT, or VF, occur in a substantial proportion of CA patients with implanted devices. However, the cause of death in a substantial proportion of CA patients is bradycardia or electromechanical dissociation rather than VAs [34].

The most common cause of SCD in CA patients has been secondary to electromechanical dissociation, resulting in pulseless electrical activity rather than lethal ventricular arrhythmias. Higher defibrillation thresholds and historically poor prognosis and life expectancy have made ICD therapy controversial [35]. The 2017 American Heart Association/ACC/HRS guideline for the management of patients with VAs and the prevention of SCD recommends individualized decision-making for both primary and secondary prevention of ICD [36].

## 4. Prognostic Significance of Arrhythmias

Heart failure often leads to clinical deterioration and cardiac dysfunction; thus, it is associated with a poor prognosis in patients with CA. According to Ashraf et al., the prognosis of CA patients with muscle dysfunction is significantly affected [18]. AF is associated with increased risk for thromboembolic events, HF, and death in patients with CA. One study demonstrated that AF was associated with a 2.5-fold increase in mortality in patients with CA. AFL and other supraventricular tachycardias in CA patients are also associated with an increased risk of thromboembolic events [18], whereas VAs have the highest risk of SCD.

Arrhythmias are associated with higher in-hospital mortality, AHF exacerbations, longer hospital stays, and higher hospital expenses in patients hospitalized for CA. All-cause death is considerably greater in patients with CA hospitalized with concomitant arrhythmias than in those without (13.9% vs. 5.3%) [5]. In both ATTR and AL CA, the onset of congestive HF caused by CA is independently associated with poor prognosis and increased mortality [5].

It should be noted that patients with AL amyloidosis have worse outcomes than patients with ATTRwt amyloidosis [37]. The prognosis of CA patients is generally poor, ranging from, on average, 6 months to several years, depending on disease subtype and stage. The presence of arrhythmias has been shown to be an important predictor of mortality. In AL amyloidosis, the survival rate of patients with AL amyloidosis is 6 months to 3 years. If it occurs in AL CA, it is associated with a poor prognosis and a high risk of death. Arrhythmias in ATTR CA are also associated with a worse prognosis and an increased risk of cardiac hospitalization and mortality [4].

AF is associated with increased morbidity and has been shown to be an independent predictor of mortality in CA patients. Loss of the atrial contribution to ventricular filling is poorly tolerated and is linked to clinical worsening and frequent hospitalizations because CA has substantial diastolic dysfunction [38]. Its presence increases the risk of intracardiac thrombus, which can lead to stroke and systemic embolism. AL amyloid appears to carry a higher risk of these complications in comparison with ATTR. The presence of atrial thrombus frequently makes it unable to use rhythm management techniques for AF [38].

In patients with ATTR-CA, AF is associated with increased left ventricular filling pressures and atrial deposition of insoluble misfolded precursor proteins [39]. Maintaining normal sinus rhythm is associated with improved survival in patients with ATTR-CA and AF. In a study of 382 patients with ATTR-CA, those with AF had a higher mortality rate compared to those without AF (69% vs. 43%) [15]. The study also found that the advanced ATTR-CA stage, higher NYHA functional class, and the absence of systemic anticoagulation were associated with increased rates of cerebrovascular accidents in patients with AF [15].

The majority of strokes in CA patients are cardio-embolic and occur in those with new AF/AFL or who are inadequately anticoagulated. Ischemic stroke can be the first presentation of CA or AF/AFL in CA patients, highlighting the importance of early detection and management of arrhythmias in this population. Given the high prevalence of arrhythmias in CA patients and their impact on morbidity and mortality, routine screening for AF and AFL is recommended in this population. Ambulatory monitoring, such as Holter monitoring or ambulatory monitors, can be used to screen for arrhythmias in CA patients [40]. Early detection and management of arrhythmias, including appropriate anticoagulation for those with AF/AFL, can help improve outcomes in CA patients [40]. The rate of thromboembolic events is as high as 5–10% amyloidosis patients, at least in patients with cardiac involvement [41]. Thromboembolic events are associated with a poor prognosis within 1 month after the event and even lower survival in the year following the event [42].

Goldsmith et al. found that patients with the highest number of VA events were more likely to die [23]. Additionally, cases of mild, life-threatening arrhythmias were reported and treated in some participants, some of whom survived six months post-SCT, emphasizing the importance of ongoing telemetric monitoring in these patients [23]. The presence of complex VAs was found to significantly influence survival, but the significance of VT in predicting SCD is uncertain. The study also showed that Holter monitoring can help assess the prognosis of AL amyloidosis patients, but its limitations should be considered; therefore, the prognostic role of each arrhythmia is different in CA patients [22].

Moving to conduction system abnormalities, it is important to note that the incidence of overt conduction disease in the setting of amyloidosis may be underestimated by studies, as SCD due to bradyarrhythmias may be the first manifestation of conduction system disease in this population [37]. A study by Reisinger et al. found that HV prolongation was common in patients with CA and was associated with subsequent SCD [30]. However, the cause of SCD in these patients was more often due to ventricular tachyarrhythmias than to complete AV block. The lack of inducibility of sustained monomorphic VT did not portend a favorable prognosis in terms of sudden death [30].

In summary, arrhythmias significantly impact patient outcomes. AF and AFL are associated with increased mortality and hospitalizations, while ventricular arrhythmias are associated with a high risk of SCD. Factors other than physical activity type have been identified as predictors of poor outcome in CA patients, including age, NYHA functional group, comorbidities such as renal dysfunction, and the presence of hypertension [7].

## 5. Treatment

Arrhythmias adversely affect the prognosis of CA patients, highlighting the importance of arrhythmic surveillance and management strategies in this population. Supraventricular arrhythmias (SVAs), such as atrial fibrillation (AF) and atrial flutter, can be managed with rate control or rhythm control strategies. Rate control is achieved with beta-blockers and/or calcium channel blockers, while rhythm control can be achieved with antiarrhythmic drugs, such as amiodarone, or AF ablation. However, the use of antiarrhythmic drugs in CA patients should be carefully considered due to the potential for drug-induced proarrhythmia and toxicity [24].

AF in CA patients should be considered differently than in other forms of HF. Caution is needed when using conventional pharmacotherapies, as they may have limited efficacy and potential complications in CA patients [13]. Rate control is the primary treatment strategy in most of the patients. Beta-blockers and non-dihydropyridine calcium channel blockers (CCBs) are the preferred (1st line) agents for rate control in CA. However, according to some researchers, non-dihydropyridine CCBs are contraindicated for their negative inotropic/chronotropic effect and the high risk of hypotension [6]. Beta-blockers may also be poorly tolerated; however, low doses may be an option to achieve rate control in AF with a rapid ventricular response [6]. It is important to note that most of the times, AF in CA patients is not related to rapid ventricular rate due to concomitant conduction system disease.

AF and CA carry a high risk of stroke, making anticoagulation recommended in these patients, regardless of the CHA2DS2-VASc score. Anticoagulation therapy is recommended to prevent thromboembolic events. Direct oral anticoagulants (DOACs), including rivaroxaban, apixaban, and dabigatran, appear to be at least as effective and safe as vitamin K-antagonists (VKAs) (coumadine, fluindione or acecoumarol) in patients with atrial arrhythmias and cardiac amyloidosis during a 2.4-year follow-up [43].

In patients with symptomatic AF despite rate control, rhythm control strategies, such as direct current cardioversion (DCCV) and antiarrhythmic drugs (AADs), may be considered [26]. However, it is important to note that the efficacy and safety of these treatments in CA patients have not been well established, and that caution should be exercised due to the potential for drug toxicity and proarrhythmic effects [9,44]. Even DCCV in CA patients is related to increased risk of complications compared to AF patients without CA. Rhythm control with AADs may be considered in certain patients, particularly those with symptomatic AF or those who are younger and have fewer comorbidities. Class Ic AADs, such as flecainide and propafenone, are effective in maintaining sinus rhythm in patients with paroxysmal AF, but their use in CA is limited. Class III AADs, such as amiodarone, sotalol, and dofetilide, are also effective in maintaining sinus rhythm and are often used in patients with persistent or long-standing AF. However, these drugs have significant side effects and require careful monitoring [40]. A safe option for AF control is the oral administration of amiodarone [6]. Digoxin remains controversial in amyloidosis due to the high risk of toxicity [6]. It is significant for patients with amyloidosis to maintain sinus rhythm by either cardioversion or ablation from the early stages of the disease in order to reduce hospitalizations and improve symptoms [6]. However, sinus rhythm may not guarantee normal contractility of the left atrium or AF relapse [6]. New therapies, such as inotersen, AG-10, and Ab-A, are being evaluated in clinical or preclinical studies for ATTR-AC, and they may change prognosis [35,45].

Atrial flutter (AFL) can also be treated with rate control and rhythm control strategies. Beta-blockers and non-dihydropyridine CCBs are the preferred agents for rate control. DCCV and AADs can also be considered for rhythm control in AFL, but the use of AADs in CA is limited [26].

Management of VAs depends on their severity and frequency. In patients with frequent or sustained VA, antiarrhythmic drugs, such as amiodarone, may be used to suppress VA. However, the use of these drugs in CA patients should be carefully monitored due to the potential for drug toxicity and proarrhythmic effects [9,46]. Conventional pharmacotherapies that are used in the treatment of VT include oral antiarrhythmic agents and beta-blockers (in small doses, if tolerated). However, beta-blockers may be detrimental to CA patients, resulting in compromise of cardiac output. Amiodarone may also result in complications when treating CA patients––namely, complete heart block and extracardiac toxicity. Its use should be cautious, especially in patients with renal impairment [21]. Chemotherapy for AL amyloidosis is mainly based on regimens used for the treatment of myeloma and is not specifically approved for AL cardiac involvement [21]. Most of these drugs have established cardiotoxic potential, with an increased risk of heart failure and arrhythmic events [21].

The management of VAs is mainly based on ICD therapy. ICDs have been shown to reduce the risk of SCD in CA patients with VAs, and, thus, they are considered the primary treatment strategy for VT and VF in CA patients. They are recommended for CA patients with a high risk of SCD due to VAs [24]. AADs can also be considered for the prevention of VT and VF in CA, but their use is limited due to the risk of adverse effects and drug interactions [26]. In a study by Lin et al., 28% of 53 patients received appropriate ICD shocks at 1 year, with patients with AL CA comprising most of those who received appropriate shocks. However, ICD therapy was not associated with improved mortality in follow-up [47]. Patients with cardiac amyloidosis frequently show worse prognosis than other types of HF and, thus, strategies for primary prevention, such as ICDs, may not help in the advanced stages [6]. For VAs, AADs are often used as adjunctive therapy to ICDs or catheter ablation. Class I AADs, such as lidocaine and mexiletine, are effective in treating VT, and they are often used in acute settings. However, these drugs have limited efficacy in preventing recurrent VT, and they can also have significant side effects. Class III AADs, such as amiodarone and sotalol, are effective in preventing recurrent VT, and they are often used as adjunctive therapy to ICDs or catheter ablation [40]. However, these drugs also have significant side effects and require careful monitoring [40].

For conduction abnormalities, pacemaker implantation (PM) is usually recommended to prevent bradycardia and syncope. The conventional strategy of pulmonary vein isolation and linear left atrial ablations may not be effective in suppressing arrhythmias in CA patients, as evidenced by the high recurrence rate after catheter ablation [16].

## 6. Device Therapy in Cardiac Amyloidosis

In addition to pharmacological, standard device therapies and other advanced non-pharmacological interventions, such as catheter ablation and cardiac resynchronization therapy (CRT), may also be considered for the management of arrhythmias in CA patients.

Conduction disturbances and bradyarrhythmias are frequent in patients with cardiac amyloidosis due to the infiltration of major conductive points of the myocardium by the amyloid fibrils. Common findings in patients’ ECG are wide QRS complexes, first-degree atrioventricular (AV) block, high-degree AV block, and progressive sinus node dysfunction, and, especially in cases of atrioventricular conduction defects, pacemaker implantation may be necessary most times [48]. Donnellan et al. [49] investigated the impact of right ventricular (RV) pacing burden and biventricular pacing on LVEF, mitral regurgitation (MR) severity, NYHA functional class, and mortality in 78 patients with ATTR (amyloidosis and transthyretin) cardiac amyloidosis and implantable devices during a mean follow-up of 42 months. The authors reported an association between higher RV pacing burden and deleterious remodeling and congestive heart failure in patients with ATTR cardiac amyloidosis. Moreover, biventricular pacing was associated with improvements in LVEF, NYHA class, and MR severity, indicating that this might be considered in these patients with an indication for pacing [49]. Preventive strategies of bradyarrhythmias and symptomatic bradycardias suggest the use prophylactic pacemaker implantation in patients with ATTR cardiac amyloidosis who present conduction disorders in the ECG, such as fascicular blocks (right bundle branch block, left bundle branch block, left anterior haemiblock, and left posterior haemiblock), first-degree AV block (PR interval ≥200 ms), or Wenckebach anterograde point ≤100 b.p.m. [50].

Screening electrophysiological studies are not indicated by guidelines in all cardiac amyloidosis patients without suggesting symptoms but with moderate conduction disturbances on the ECG. In these patients, ECG Holter monitoring could be a useful diagnostic tool in the further investigation of the arrhythmias [51].

Sudden death is common among patients with end-stage cardiac amyloidosis, with pulseless electrical activity and/or agonal bradycardia traditionally considered as the most frequently documented terminal events [52]. The use of implantable cardioverter-defibrillators (ICDs) as a primary prevention strategy might not be beneficial in these cases. However, in amyloidosis cardiomyopathy, especially in AL CA, arrhythmic sudden death due to ventricular arrhythmias may be more frequent than commonly believed, and the use of ICDs in these patients could be beneficial. Even a traditional significant limitation for ICD placement, the poor patients’ prognosis, could be overcome with modern targeted therapies. Once a patient’s prognosis remains poor, with less than a 12 month survival after diagnosis, the implantation of ICDs is contraindicated [53]. During recent years, several retrospective analyses have investigated the outcomes of ICDs implantation for primary and secondary prevention in patients with cardiac amyloidosis and tachyarrhythmias, including non-sustained VT, VT, or cardiac arrest (pulseless VT, ventricular fibrillation) [1,47,54,55,56,57,58]. Some of these studies have demonstrated a high rate of appropriate discharges. However, overall mortality in cardiac amyloidosis is similar between patients with an ICD discharge and those without [47,54]. Due to the lack of evidence of a survival benefit in patients after ICD implantation for primary prevention, at least in the existing data, their routine use for primary prevention in cardiac amyloidosis is yet not recommended [47].

**Figure 1:**
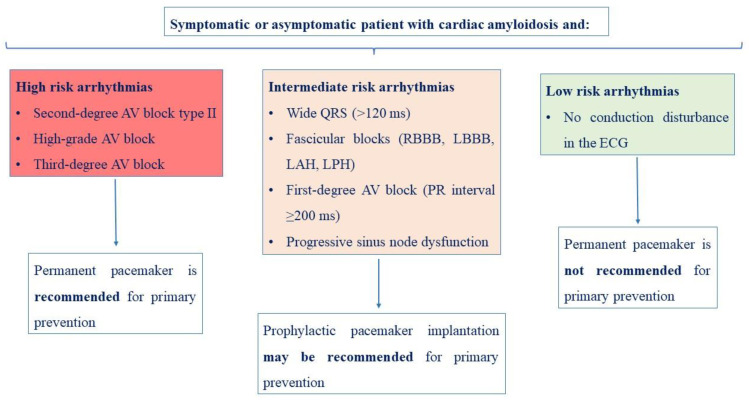
Proposed algorithm for permanent pacemaker implantation in patients with cardiac amyloidosis and arrythmias [20,31,37,51,52,53,58].

**Figure 2:**
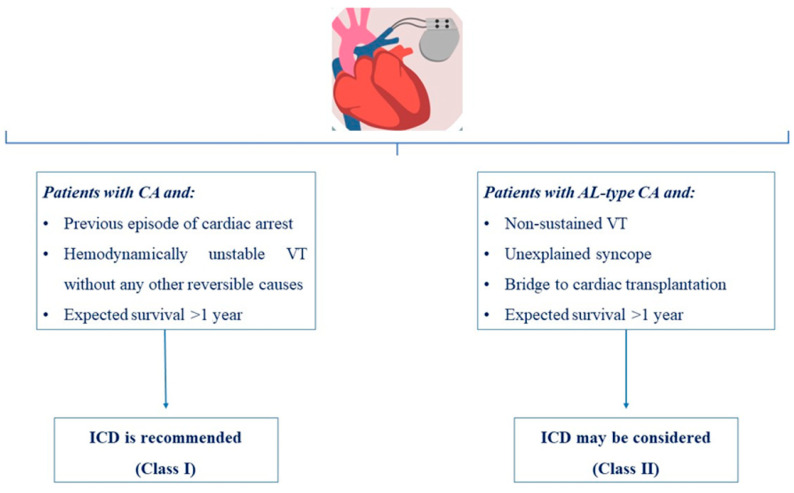
Proposed algorithm for implantable cardioverter-defibrillator (ICD) in patients with cardiac amyloidosis and arrythmias [20,37,54].

The choice between a conventional pacemaker and an ICD remains controversial in patients with cardiac amyloidosis. Usually, an individual decision should be made based on a patient’s characteristics, the type of conduction disturbance or the arrhythmia, and the risk stratification according to the most updated guidelines [19,36]. Proposed algorithms for the implantation of a permanent pacemaker and/or an ICD in patients with cardiac amyloidosis and arrythmias are presented in Figure 1 and Figure 2, respectively.

## 7. Clinical Management

**Figure 3:**
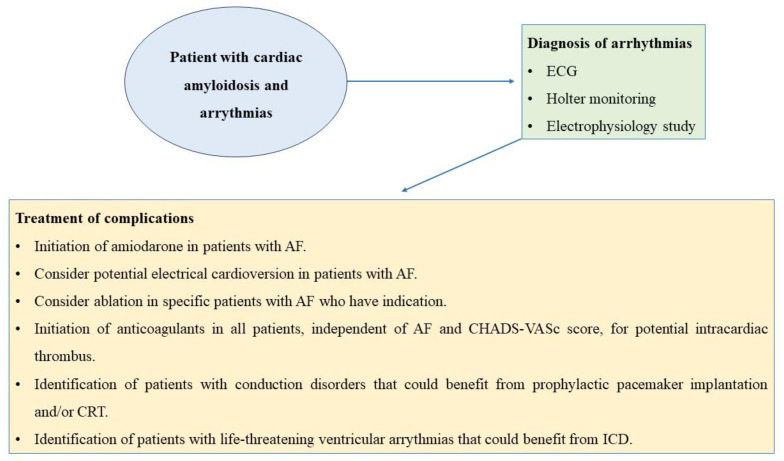
Clinical management for patients with cardiac amyloidosis and arrythmias [20,37,52,54].

Clinical management of patients with cardiac amyloidosis and arrythmias is considered as a significant variable of prognostication and survival. Early diagnosis and immediate treatment and/or supportive therapy should be implemented. A proposed algorithm of clinical management of CA patients with arrythmias is demonstrated in Figure 3.

## 8. Conclusions

Heart rhythm disturbances are quite common in cardiac amyloidosis, varying in prevalence, type, severity, and clinical manifestation according to CA subtype and individual patient disease status. Atrial fibrillation is the most frequent supraventricular arrhythmia in this group of patients, and clinical management remains a major challenge. Bradyarrhythmias are also common, particularly atrioventricular conduction defects, and appropriate treatment with permanent pacemakers when applicable should not be delayed. Ventricular arrhythmias management is mostly challenging, especially regarding beneficial ICD implantation. Truly effective treatments must be supported by careful consideration involving nuances such as overall health status, disease stage, disease severity, disease type, and associated complicating co-morbidities. CA prognosis could be improved by early detection and timely treatment of arrhythmias, and a multidisciplinary approach is often mandatory in this setting.

## Data Availability

Not applicable.
